# Gender-specific associations of serum sex hormone-binding globulin with features of metabolic syndrome in children

**DOI:** 10.1186/s13098-016-0134-8

**Published:** 2016-03-08

**Authors:** Nasser M. Al-Daghri, Nasiruddin Khan, Shaun Sabico, Omar S. Al-Attas, Majed S. Alokail, Sudhesh Kumar

**Affiliations:** Prince Mutaib Chair for Biomarkers of Osteoporosis, Biochemistry Department, College of Science, King Saud University, P.O. Box 2455, Riyadh, 11451 Saudi Arabia; Biomarkers Research Program, Biochemistry Department, College of Science, King Saud University, Riyadh, 11451 Saudi Arabia; Division of Metabolic and Vascular Research, University of Warwick, Coventry, CV2 2DX UK

**Keywords:** Sex hormone binding globulin, Metabolic syndrome, Arab children

## Abstract

**Background:**

Sex hormone-binding globulin (SHBG) has been proposed as a biomarker of MetS in children and adults. We aim to determine the associations of SHBG with components of MetS in children from the Middle-East where the prevalence of MetS are on the rise.

**Methods:**

In this cross-sectional study, a total of 509 randomly selected school children (226 boys and 283 girls) aged 12–16 years were recruited. Fasting blood glucose and lipid profile were determined using routine laboratory procedures. Serum SHBG is measured with Cobas e411 using an electrochemiluminescence immunoassay. The modified definition of ATP-III (NHANES III) was used for the diagnosis of MetS.

**Results:**

Among 509 children, 23.4 % had metabolic syndrome. Boys had a significantly higher waist circumference and systolic blood pressure (p < 0.032, 0.024, respectively) than girls, while levels of glucose (p < 0.029), and SHBG (p < 0.003) were significantly higher in girls than in boys. In overall population, a significant inverse correlation of SHBG level with age, BMI, systolic blood pressure and triglycerides and a significant direct correlation between SHBG level and HDL-c was exhibited. Children with the lowest tertile of serum SHBG had significantly higher prevalence of MetS (p < 0.05). ROC curve shows SHBG level as more sensitive marker of MetS in boys (AUC = 0.70, p < 0.001).

**Conclusions:**

Serum SHBG is significantly more sensitive in identifying MetS in boys, not girls, indicating gender dimorphism.

**Electronic supplementary material:**

The online version of this article (doi:10.1186/s13098-016-0134-8) contains supplementary material, which is available to authorized users.

## Background

Metabolic syndrome (MetS) is an assembly of several cardiometabolic risk factors (including central obesity, insulin resistance, dyslipidemia and hypertension) that may predispose towards the development of type 2 diabetes mellitus (T2DM) and cardiovascular diseases (CVDs) [1–4].

In recent years, there has been a great concern about the presence of obesity and metabolic syndrome in children. A recent systematic review that included 378 studies published since 2003 and, depending on different classifications, showed the median prevalence of MetS in children populations as 3.3 % (range 0–19.2 %), in overweight children 11.9 % (range 2.8–29.3 %), and in obese children 29.2 % (range 10–66 %) [5].

In the past few decades, Saudi Arabia has shown a very rapid development at both the economic and social fields. This change has brought about a shift of traditional Arabian diet to a more western dietary pattern predisposing adults and children to obesity, T2DM, and hypertension [6–9]. The latest national data revealed that the rates of overweight and obesity among Saudi school children have reached 21 and 9.3 %, respectively [10]. Taha et al. demonstrated that obese children and adolescents have multiple risk factors associated with MetS [11].

Sex hormone-binding globulin (SHBG) is a circulating glycoprotein involved in the transportation of sex steroids and is mainly synthesized by the liver [12, 13]. An association between SHBG levels with MetS have been observed in both men and women [14, 15]. Thus there are studies showing strong association of low levels of SHBG with obesity [16], insulin resistance [17] and MetS in men, women [18] and in pre-pubertal children [19]. Moreover, lipid profiles, such as low HDL-C and high triglycerides (TG) are associated with low SHBG level in the adult population [20, 21].

There are numerous studies in children and adolescents relating diabetes (types 1 and 2), cardiovascular risk factors and prevalence of MetS from different parts of the Saudi Arabia [9, 11, 22]. While previous researches have already been conducted on the role of SHBG and MetS even in children, there is limited data available on specific age-groups as well as the existence of gender dimorphism [23–27]. Thus, the main objective of our present study was to investigate the relation of SHBG levels and components of MetS and its potential as an early biomarker for MetS in Arab boys and girls 12–16 years of age.

## Patients and methods

### Subjects

The sample population of this cross-sectional study included 509 apparently healthy school children (226 boys and 283 girls), aged 12–16 years. Children with chronic diseases, such as type 1 diabetes mellitus (T1DM) and asthma, as well as those on medications that might influence the metabolic parameters of interest, were excluded. Subjects were recruited randomly from different Primary Health Care Centers (PHCC) across Riyadh as previously described [28]. Each participating subject submitted a general questionnaire containing demographic, past and present medical history, as well as diet information from the food frequency questionnaire. No expatriates will be included in the conduct of this study. All participants provided written informed consent prior to inclusion in the study. Approval was obtained from the Ethics Committee of the College of Science Research Center, King Saud University, Riyadh, Saudi Arabia.

### Anthropometric measurements

With parents’ written consent and oral ascent from the children, subjects were invited to their respective PHCC in a 10-h overnight fasted state. Anthropometric measurements included height (cm), weight (cm), waist (cm) and hip (cm) circumferences and were taken and noted by trained nurses. Systolic and diastolic blood pressures were taken using pediatric cuffs appropriate to the subject sizes and were taken twice with a 15-min interval. The average of both readings was recorded. Body mass index (BMI) was calculated as weight (kg) over height in squared meters (m^2^).

### Biochemical measurements

Fasting blood samples were collected and transferred immediately to a non-heparinized tube for centrifugation. Collected sera were then transferred into a pre-labeled plain tube, stored on ice and delivered to the Biomarkers Research Program (BRP) laboratory, King Saud University, Riyadh, Saudi Arabia, on the same day of collection for immediate storage in a −20 °C freezer for immediate analyses. The blood samples were analyzed for fasting glucose and lipid profile including HDL-cholesterol, using a chemical analyzer (Konelab, Espoo, Finland). Serum SHBG was measured with a Roche Elecsys modular analytics Cobas e411 using an electrochemiluminescence immunoassay [ECLIA] (Roche Diagnostics, GmbH, Mannheim, Germany). The lower detection limit of this assay was 0.35 nmol/L and the intra-assay CV is 2.6–5.6 %.

### Definition of metabolic syndrome

The pediatric definition of MetS by de Ferranti et al., which is analogous to the criteria by the Third Report of the Adult Treatment Panel (ATPIII, NHANES III), was used for this study [29]. MetS was defined as the presence of three out of five abnormalities: hypertriglyceridemia ≥1.1 mM; low HDL-cholesterol (HDL-C) <1.3 Mm (boys aged 15–19 years, <1.17 mM); high fasting glucose ≥6.1 mM; central obesity >75th percentile for age and gender; and hypertension >90th percentile for age, gender and height.

### Data analyses

Data were analyzed using SPSS (version 16.5 Chicago, IL, USA). Continuous data were presented as mean ± standard deviation (SD). Categorical data were presented as frequencies and percentages (%). All continuous variables were checked for normality using the Kolmogorov–Smirnov test. Independent Student *t* test and Mann–Whitney U test were used for significance for Gaussian and Non-Gaussian variables. Triglycerides was log-transformed prior to all parametric analyses. Bivariate correlations and linear regressions were done to determine associations between SHBG and cardiometabolic parameters. Stepwise linear regression was done in all groups and by gender to determine significant predictors using SHBG as dependent variable and the cardiometabolic parameters assessed as independent variables. Area under the curve (AUC) was done to determine sensitivity of SHBG in predicting MetS and its p value was adjusted for multiple comparisons (Bonferroni corrected p value = 0.005). All tests were twotailed and p values set on thresholds required to achieve a type 1 error of 0.05. p value < 0.05 was considered statistically significant.

## Results

The anthropometric and biochemical variables of the 509 individuals are shown in Table 1. Based on ATPIII criteria, 23.4 % had MetS. A significant difference in control was observed with MetS children having a lower age (p < 0.001), BMI (p < 0.004), waist circumference (p < 0.009), systolic and diastolic blood pressure (p < 0.001, 0.001, respectively), total cholesterol (p < 0.001), glucose, HDL-C and TG (p < 0.000 for all) as well a significantly lower SHBG level (p < 0.006). Characteristics of the control group according to sex is presented in Additional file 1: Table S1.

**Table 1:** Descriptive statistics of clinical and metabolic characteristics of subject studied (control vs. metabolic syndrome)

Parameters	Control	Metabolic syndrome	p value
N (boys/girls)	390 (170/220)	119 (56/63)	
Age (years)	13.95 ± 1.18	14.39 ± 1.23	*0.001*
BMI (kg/m^2^)	21.69 ± 4.65	23.15 ± 4.36	*0.004*
Waist circumference (cm)	62.07 ± 20.57	23.15 ± 24.13	*0.009*
Hip circumference (cm)	76.89 ± 26.51	81.99 ± 28.27	0.076
Systolic blood pressure (mmHg)	117.81 ± 12.88	127.84 ± 16.53	*0.000*
Diastolic blood pressure (mmHg)	69.50 ± 11.34	74.98 ± 13.86	*0.000*
Total cholesterol (mmol/l)	3.80 ± 0.93	4.13 ± 1.05	*0.001*
Glucose (mmol/l)	4.95 ± 0.87	5.55 ± 0.98	*0.000*
HDL-cholesterol (mmol/l)	0.96 ± 0.22	0.91 ± 0.22	*0.000*
Triglycerides (mmol/l)^a^	1.05 ± 0.47	1.52 ± 0.59	*0.000*
Sex-hormone binding globulin (nmol/l)	60.47 ± 29.43	51.76 ± 30.03	*0.006*

Data presented as mean ± standard deviation for normal continuous variables

Significant p-values (< 0.05) were italicized

^a^Non-Gaussian distribution

Boys with MetS, had significantly higher waist circumference and systolic blood pressure (p < 0.032, 0.024, respectively) than girls, while the levels of glucose (p < 0.029) and SHBG (p < 0.003) were significantly higher in girls than boys (Table 2).

**Table 2:** Clinical and metabolic characteristics of the metabolic syndrome groups (boys vs. girls)

Parameters	Boys	Girls	p value
N	56	63	
Age (years)	14.39 ± 1.30	14.39 ± 1.17	0.986
BMI (kg/m^2^)	23.93 ± 4.68	22.54 ± 4.03	0.107
Waist circumference (cm)	73.15 ± 27.52	63.52 ± 19.81	*0.032*
Hip circumference (cm)	85.11 ± 31.04	79.23 ± 25.52	0.268
Systolic blood pressure (mmHg)	131.62 ± 17.71	124.57 ± 14.84	*0.024*
Diastolic blood pressure (mmHg)	74.26 ± 14.07	75.59 ± 13.78	0.619
Total cholesterol (mmol/l)	4.24 ± 0.82	4.03 ± 1.22	0.291
Glucose (mmol/l)	5.34 ± 1.06	5.73 ± 0.86	*0.029*
HDL-cholesterol (mmol/l)	0.87 ± 0.23	0.94 ± 0.20	0.081
Triglycerides (mmol/l)^a^	1.54 ± 0.58	1.50 ± 0.61	0.777
Sex-hormone binding globulin (nmol/l)	42.34 ± 23.32	58.15 ± 30.15	*0.003*

Data presented as mean ± standard deviation for normal continuous variables denotes

Significant p-values (< 0.05) were italicized

^a^Non-Gaussian distribution

Table 3 represents the correlation of SHBG with various anthropometric and biochemical parameters in all subjects as well as in boys and girls. A significant inverse correlation of SHBG level with age, BMI, waist circumference, systolic blood pressure and TG (p < 0.01 for all) were observed in the overall population, while a significant direct correlation between SHBG level and HDL-C were also exhibited in overall group. Importantly, the gender stratification showed significant correlations of SHBG level with more parameters of MetS in boys than girls. Thus, in boys, significant inverse correlations of SHBG levels were observed with age, BMI, TG, systolic blood pressure (p < 0.01 all), and waist circumference (p < 0.05), while in girls correlations of SHBG were exhibited with BMI, systolic blood pressure (p < 0.01), and TG (p < 0.05). A significant direct correlation between SHBG level and HDL-C was noticed in both boys and girls.

**Table 3:** Correlation coefficients of SHBG with metabolic parameters for all subjects, boys and girls

Parameters	All subjects	Boys	Girls
N	509	265	244
Age (years)	−0.224**	−0.311**	−0.123
BMI (kg/m^2^)	−0.399**	−0.390**	−0.399**
Waist circumference (cm)	−0.104*	−0.128*	−0.073
Hip circumference (cm)	−0.068	−0.107	−0.027
Systolic blood pressure (mmHg)	−0.319**	−0.339**	−0.284**
Diastolic blood pressure (mmHg)	−0.045	−0.087	0.009
Total cholesterol (mmol/l)	−0.007	−0.061	0.039
Glucose (mmol/l)	−0.076	−0.046	−0.127
HDL-cholesterol (mmol/l)	0.280**	0.311**	0.236**
Triglycerides (mmol/l)^a^	−0.206**	−0.242**	−0.166*

Data presented as coefficient (R)

* p < 0.05, ** p < 0.01

^a^Denotes log transformed

Stepwise linear regression showed that BMI, systolic and diastolic blood pressure, age and HDL-cholesterol cumulatively predicted 26.6 % (Adjusted R^2^ 0.256; p < 0.001) of the variances perceived in circulating SHBG in all subjects. Splitting by sex revealed that in boys, the significant predictors for SHBG include BMI, systolic blood pressure and age (R^2^ 0.31; p < 0.001) while in girls, the significant predictors were BMI and blood pressure (systolic and diastolic) (R^2^ 0.19; p < 0.001) (not shown in tables).

**Fig. 1:**
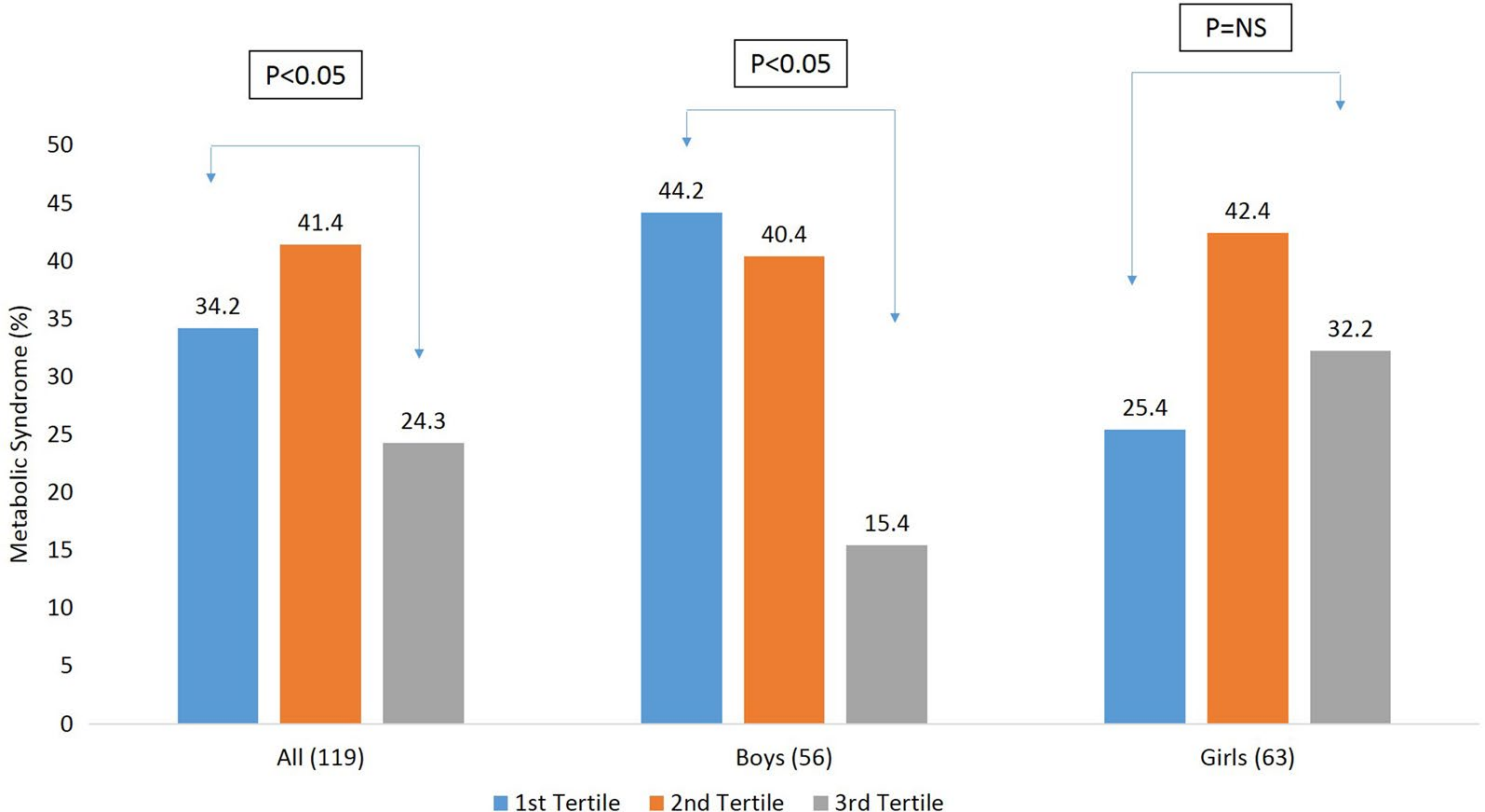
Prevalence of MetS based on tertiles of SHBG level. *NS* non-significant

**Fig. 2:**
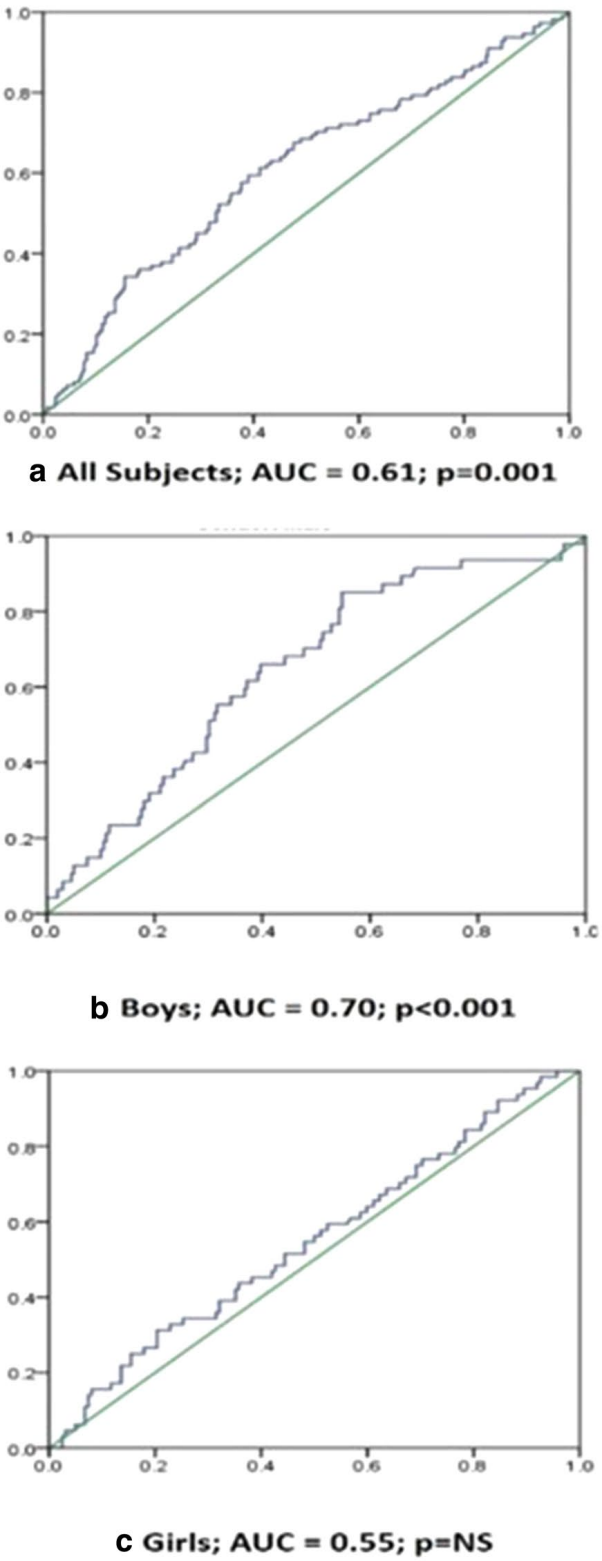
Receiver operating characteristic (ROC) analysis curve using SHBG serum levels as a predictor of MetS

Based on tertiles of SHBG levels, a decrease in the prevalence of MetS was observed in the overall and MetS group (Fig. 1). However, the effect was more pronounced and uniform in boys, showing the highest prevalence of MetS with the lowest tertile of SHBG levels, than girls. The ROC curves (Fig. 2) show that SHBG levels is a more sensitive marker of MetS, with significant values in overall subjects (AUC = 0.61, p < 0.001) and boys (AUC = 0.70, p < 0.001), but not in girls (AUC = 0.55 p = NS).

## Discussion

To our knowledge, this is the first study to report the gender dimorphic sensitivity of serum SHBG in identifying Arab children with MetS. The prevalence of MetS in our cohort was 23.4 % and differences in boys and girls were observed in the associations of cardiometabolic parameters to circulating SHBG. A significant inverse correlation of SHBG level with age, BMI, waist circumference, systolic blood pressure, and TG, and a positive correlation of HDL-C was observed. SHBG was associated in determining full MetS in boys, not girls.

In the year 2010, a cross-sectional observational study, based on the same definition to define MetS as the present one, including a total of 1231 Saudi children and adolescents demonstrated that the prevalence of MetS manifestations among Arab children is extremely high with overall prevalence of complete MetS as high as 9.4 % [22]. Although not a representative of the Saudi children population, this study demonstrated an alarmingly high prevalence of MetS in the Arab children, as high as 23.4 %. The main causative factors, such as overweight and obesity, which lead to increased prevalence of MetS and other non-communicable diseases in both children and adults, have been mainly contributed by unhealthy dietary patterns, low physical activity and, to some extent, the social norms of the society [30–33].

Important hormonal changes takes place during puberty age in boys and girls. Pinkney and colleagues demonstrated that SHBG level keeps increasing in the earlier stage of childhood with a gradual decline as they reach their age at puberty, and that this effect is predominant in boys [34]. Moreover, in a cross-sectional study performed by Garces and colleagues including pubertal children (age: 12–15 years), a significantly higher SHBG level was demonstrated in girls than boys, reinforcing that the sex and growth hormone variations during puberty manifests earlier in girls than boys of the same age. In addition, a significant inverse relation between testosterone and SHBG level was shown in boys, with a gradual increase with age, while this effect was not uniform in girls with SHBG levels not showing significant differences based on age groups [35]. This study includes boys and girls with advancing age and puberty and supports the above results, showing significantly lower SHBG levels in boys than girls in MetS.

The correlation results observed in our study are interesting in the way that they are associated with cardiometabolic risk factors in boys and girls. These results demonstrating an inverse correlation between age and SHBG level with a significant value in boys supports the findings of de Oya et al. [25], Pinkney et al. [34] and Sorensen et al. [36].

Insulin down-regulates hepatic SHBG production [19], while an inverse correlation between BMI and insulin sensitivity has been shown in various studies in obese children and adolescents [37, 38]. Our study demonstrating an inverse correlation between BMI and SHBG levels is also in accordance with previous investigations in children [39, 40]. Krishnasamy and colleagues demonstrated an inverse relation between SHBG levels and waist circumference, as well as body mass index percentile in children at risk for MetS [23]. Our study partially supports the above results showing a significant inverse correlation between SHBG levels and waist circumference but only in boys. There is evidence showing an inverse association between SHBG, BP and TG in adults and children [40, 41]. The studies also observed a direct association of SHBG with HDL-c level. Our study supports the above results showing a significant inverse correlation between SHBG, systolic BP and TG levels and a direct relation between SHBG with HDL-c levels in the MetS group including boys and girls. The lowest tertile of SHBG level showed a higher prevalence of MetS in boys, with a uniform inverse trend than girls. The possible explanation for this trend may the correlation of SHBG level with more MetS components as compared to girls. The ROC analysis also supports the above trend which shows SHBG as a clearer predictor of MetS components in boys than girls.

## Conclusions

In conclusion, our study showed that SHBG is a sensitive predictor of metabolic syndrome in boys and not girls. This study is limited with its cross-sectional study design which prevents causality. The study is nevertheless one of the few to demonstrate significant gender dimorphism of SHBG’s influence in children of a specific age-group. The use of SHBG as a useful marker to identify boys with increased cardiometabolic risk is promising.

## Additional file

10.1186/s13098-016-0134-8 Characteristics of the control group (boys vs. girls).

## Abbreviations

AUC: area under the curve
BMI: body mass index
CVD: cardiovascular diseases
HDL-C: hdl-cholesterol
MetS: metabolic syndrome
NS: non-significant
PHCC: Primary Health Care Center
SHBG: sex hormone-binding globulin
T2DM: type 2 diabetes mellitus
TG: triglycerides
