# Supplementary material for: Gender-specific associations of serum sex hormone-binding globulin with features of metabolic syndrome in children
Source: Diabetol Metab Syndr. 2016 Mar 8;8:22. doi: 10.1186/s13098-016-0134-8 (PMC4784466; doi:10.1186/s13098-016-0134-8)
Supplement: Supplementary file 1 — 10.1186/s13098-016-0134-8 Characteristics of the control group (boys vs. girls). [file 13098_2016_134_MOESM1_ESM.docx]

**Table S1**. Characteristics of the Control Group (Boys vs. Girls)

| Parameters | Boys | Girls | P-Value |
| --- | --- | --- | --- |
| N | 209 | 181 |  |
| Age (years) | 14.0 ± 1.1 | 13.8 ± 1.2 | 0.08 |
| BMI (kg/m^2^) | 22.1 ± 4.9 | 21.2 ± 4.3 | 0.05 |
| Waist Circumference (cm) | 61.0 ± 22.4 | 63.3 ± 18.1 | 0.29 |
| Hip circumference (cm) | 75.0 ± 28.8 | 79.1 ± 23.4 | 0.13 |
| Systolic blood pressure (mmHg) | 118.9 ± 14.0 | 116.4 ± 11.2 | 0.07 |
| Diastolic blood pressure (mmHg) | 70.6 ± 11.8 | 68.2 ± 10.7 | 0.05 |
| Total Cholesterol (mmol/l) | 3.8 ± 0.8 | 3.8 ± 1.0 | 0.56 |
| Glucose (mmol/l) | 4.9 ± 0.8 | 5.0 ± 0.8 | 0.77 |
| HDL-Cholesterol (mmol/l) | 0.96 ± 0.2 | 0.97 ± 0.2 | 0.36 |
| Triglycerides (mmol/l) # | 1.1 ± 0.4 | 1.0 ± 0.5 | 0.54 |
| Sex-Hormone Binding Globulin (nmol/l) | 58.3 ± 28.8 | 62.0 ± 28.5 | 0.22 |

**Note:** Data presented as mean ± standard deviation for normal continuous variables; # denotes non-Gaussian distribution; p-value significant at p<0.05
